# Reactive astrogliosis is associated with higher cerebral glucose consumption in the early Alzheimer’s *continuum*

**DOI:** 10.1007/s00259-022-05897-4

**Published:** 2022-07-18

**Authors:** Gemma Salvadó, Marta Milà-Alomà, Mahnaz Shekari, Nicholas J. Ashton, Grégory Operto, Carles Falcon, Raffaele Cacciaglia, Carolina Minguillon, Karine Fauria, Aida Niñerola-Baizán, Andrés Perissinotti, Andréa L. Benedet, Gwendlyn Kollmorgen, Ivonne Suridjan, Norbert Wild, José Luis Molinuevo, Henrik Zetterberg, Kaj Blennow, Marc Suárez-Calvet, Juan Domingo Gispert, on behalf of the ALFA Study

**Affiliations:** 1 Barcelonaβeta Brain Research Center (BBRC), Pasqual Maragall Foundation, C/ Wellington, 30, 08005 Barcelona, Spain; 2 IMIM (Hospital del Mar Medical Research Institute), Barcelona, Spain; 3 Centro de Investigación Biomédica en Red de Fragilidad Y Envejecimiento Saludable (CIBERFES), Instituto de Salud Carlos III, Madrid, Spain; 4 Universitat Pompeu Fabra, Barcelona, Spain; 5 Department of Psychiatry and Neurochemistry, Institute of Neuroscience and Physiology, The Sahlgrenska Academy, University of Gothenburg, Gothenburg, Sweden; 6 Wallenberg Centre for Molecular and Translational Medicine, Department of Psychiatry and Neurochemistry, Institute of Neuroscience and Physiology, the Sahlgrenska Academy at the University of Gothenburg, Gothenburg, Sweden; 7 Institute of Psychiatry, King’s College London, Maurice Wohl Clinical Neuroscience Institute, Psychology & Neuroscience, London, UK; 8 NIHR Biomedical Research Centre for Mental Health & Biomedical Research Unit for Dementia at South London & Maudsley NHS Foundation, London, UK; 9 Centro de Investigación Biomédica en Red Bioingeniería, (CIBER-BBN), Biomateriales Y Nanomedicina, Barcelona, Spain; 10 Nuclear Medicine Department, Hospital Clínic Barcelona, Barcelona, Spain; 11 Translational Neuroimaging Laboratory, McGill Centre for Studies in Aging, McGill University, Montreal, QC Canada; 12 Roche Diagnostics GmbH, Penzberg, Germany; 13 Roche Diagnostics International Ltd, Rotkreuz, Switzerland; 14 H. Lundbeck A/S, Copenhagen, Denmark; 15 Clinical Neurochemistry Laboratory, Sahlgrenska University Hospital, Mölndal, Sweden; 16 Department of Neurodegenerative Disease, UCL Institute of Neurology, Queen Square, London, UK; 17 UK Dementia Research Institute at UCL, London, UK; 18 Hong Kong Center for Neurodegenerative Diseases, Hong Kong, China; 19 Servei de Neurologia, Hospital del Mar, Barcelona, Spain

**Keywords:** Glia, Gliosis, Astroglia, Reactive astrocyte, Glucose metabolism, Glucose consumption

## Abstract

**Purpose:**

Glial activation is one of the earliest mechanisms to be altered in Alzheimer’s disease (AD). Glial fibrillary acidic protein (GFAP) relates to reactive astrogliosis and can be measured in both cerebrospinal fluid (CSF) and blood. Plasma GFAP has been suggested to become altered earlier in AD than its CSF counterpart. Although astrocytes consume approximately half of the glucose-derived energy in the brain, the relationship between reactive astrogliosis and cerebral glucose metabolism is poorly understood. Here, we aimed to investigate the association between fluorodeoxyglucose ([^18^F]FDG) uptake and reactive astrogliosis, by means of GFAP quantified in both plasma and CSF for the same participants.

**Methods:**

We included 314 cognitively unimpaired participants from the ALFA + cohort, 112 of whom were amyloid-β (Aβ) positive. Associations between GFAP markers and [^18^F]FDG uptake were studied. We also investigated whether these associations were modified by Aβ and tau status (AT stages).

**Results:**

Plasma GFAP was positively associated with glucose consumption in the whole brain, while CSF GFAP associations with [^18^F]FDG uptake were only observed in specific smaller areas like temporal pole and superior temporal lobe. These associations persisted when accounting for biomarkers of Aβ pathology but became negative in Aβ-positive and tau-positive participants (A + T +) in similar areas of AD-related hypometabolism.

**Conclusions:**

Higher astrocytic reactivity, probably in response to early AD pathological changes, is related to higher glucose consumption. With the onset of tau pathology, the observed uncoupling between astrocytic biomarkers and glucose consumption might be indicative of a failure to sustain the higher energetic demands required by reactive astrocytes.

**Supplementary Information:**

The online version contains supplementary material available at 10.1007/s00259-022-05897-4.

## Background

Glial activation is one of the early mechanisms altered in Alzheimer’s disease (AD), probably in response to amyloid-β (Aβ) deposition [1–5]. Contrary to what was previously thought, glial activation has emerged as an active contributor to AD evolution [6], which may influence not only the propagation of the main pathological hallmarks but also the clinical evolution of cognitive disturbances in patients [7, 8]. Reactive astrocytes can affect several different phenotypes and trigger morphological, functional and molecular changes [9, 10]. This process, named reactive astrogliosis, can be investigated with fluid biomarkers such as glial fibrillary acidic protein (GFAP), S100B, and chitinase-3-like protein 1 (YKL-40) (see for a review [11]).

GFAP is an astrocytic intermediate filament protein, mainly expressed in the brain (https://www.proteinatlas.org/ENSG00000131095-GFAP/tissue), and variations in its immunoreactivity reflect changes in astrocyte cytoskeleton [10, 11]. GFAP can be measured in CSF and, more recently, also in blood, which allows more convenient assessments. Several studies in symptomatic AD showed an increase in CSF or blood GFAP [12–17], although some studies did not find them [18, 19]. Plasma GFAP has showed interesting characteristics compared to its CSF counterpart, as it is elevated already in preclinical AD [20, 21]. In a previous multi-centric study, we showed that plasma GFAP has an early increase in the preclinical stage of Alzheimer’s, which appears to be driven by Aβ pathology [22]. Further, plasma GFAP has also been observed to be elevated in other neurological conditions presumably as a sign of astrocytic response to brain injury [23–25].

[^18^F]fluorodeoxyglucose ([^18^F]FDG) is a glucose analog labeled with a positron emitter isotope (^18^F) that allows measurement of regional cerebral glucose consumption using positron emission tomography (PET). In symptomatic stages of AD, [^18^F]FDG uptake is typically reduced in temporo-parietal brain regions, probably reflecting, at least in part, synaptic dysfunction and neuronal loss [26, 27]. As such, it is considered to be a well-established marker of neurodegeneration in AD [28]. Conversely, higher [^18^F]FDG uptake has been reported in cognitively unimpaired individuals with positive AD biomarkers [29–31], possibly related to neuroinflammatory processes [32]. More specifically, we have shown that higher [^18^F]FDG uptake in preclinical AD is associated with higher levels of core AD biomarkers and other markers related to neuroinflammation [31].

However, the specific source of the [^18^F]FDG-PET signal has been the focus of extensive debate. Traditionally, it has been only attributed to neuronal uptake, with hypometabolism being considered as a direct index of neuronal dysfunction or death. But other brain cells are also metabolically active, including astrocytes. Based on the astrocyte-neuron lactate shuttle hypothesis, the activation of the glutamate transporter 1 (GLT-1) acts as a trigger for glucose uptake by astrocytes. This hypothesis postulates that, under physiologic conditions, neurons use glucose for the production of ATP. At the same time, in astrocytes, glucose is predominantly used to produce lactate, which is then released and taken up by neurons as an additional source of energy [33, 34]. As consequence, astrocytes might account for a substantial proportion of cerebral glucose utilization [35–38]. In this line, in two studies with rodents, the pharmacological modulation of the astrocytic GLT-1 activity was linked to the [^18^F]FDG PET signal, suggesting a direct association between astrocytic activity and [^18^F]FDG uptake [39, 40]. Nonetheless, the interpretation of these results needs further confirmation, especially on its translation to humans (see [41] for a comment).

For this reason, the investigation of the association between astrogliosis and glucose consumption in humans is relevant at this point. Thus, in this work, we studied the associations between plasma and CSF GFAP, markers of astroglial reactivity, with [^18^F]FDG uptake in the brain of cognitively unimpaired individuals, many of them early in the Alzheimer’s *continuum*. As it has been hypothesized that the evolution of glial biomarkers is not monotonic in the course of AD [42, 43], we also investigated interactions as a function of AT stages [44].

## Methods

### Participants

All participants included in this study were part of the ALFA + cohort, a nested study of the ALFA for ALzheimer's and FAmilies) parent cohort [45]. The ALFA cohort was established as a research platform to characterize preclinical AD in 2743 cognitively unimpaired individuals, aged between 45 and 75 years old, and enriched for family history of sporadic AD. From this parent cohort, 419 ALFA + participants were selected to be preferentially *APOE-ε4* carriers and/or to be adult children of AD patients. These participants underwent a more comprehensive evaluation including a lumbar puncture and an Aβ and [^18^F]FDG PET. For this study, we included the first 327 consecutive participants that had usable CSF and [^18^F]FDG PET data acquired within less than a year.

The ALFA + study (ALFA-FPM-0311) was approved by the Independent Ethics Committee “Parc de Salut Mar,” Barcelona, and registered at Clinicaltrials.gov (Identifier: NCT02485730). All participants signed the study’s informed consent form that had also been approved by the Independent Ethics Committee “Parc de Salut Mar,” Barcelona. The study protocol conformed to the principles set out in the WMA Declaration of Helsinki and the Department of Health and Human Services Belmont Report.

### Fluid biomarkers

Plasma and CSF samples were analyzed at the Clinical Neurochemistry Laboratory, Sahlgrenska University Hospital, Mölndal, Sweden, by board-certified laboratory technicians who were blinded to other data. The blood and CSF sample collection and the processing procedure have been described previously [1, 46]. Both plasma and CSF GFAP were quantified on a Simoa HD-X (Quanterix, Billerica, MA, USA) using the commercial single-plex assay (#102,336). CSF phosphorylated tau (p-tau) and total tau (t-tau) were measured using the electrochemiluminescence Elecsys® Phospho-Tau (181P) CSF and Total-Tau CSF immunoassays, respectively, and on a fully automated cobas e 601 instruments (Roche Diagnostics International Ltd) [47]. Aβ42 and Aβ40 were measured with the exploratory Roche NeuroToolKit immunoassays (Roche Diagnostics International Ltd) on a cobas e 411 analyzer or cobas e 601 modules.

Individuals were classified by AT groups using very sensitive in-house cutoffs of CSF Aβ42/40 ratio (A + : < 0.071) and p-tau (T + : > 24 pg/mL), previously validated [1]. A-T + participants (i.e., non-AD pathological changes; *n* = 12) were excluded as they do not belong to the AD *continuum* [28]. The extreme values of each biomarker, defined as those that fell outside three times the interquartile range above the third quartile or below the first quartile, were also removed (one CSF GFAP measurement). One subject was also excluded due to an extreme global [^18^F]FDG uptake value following the same criterion.

### Image acquisition and processing

All participants had a T1-weighted MRI, an Aβ [^18^F]flutemetamol PET, and a [^18^F]FDG PET scans acquired within one year of the GFAP determinations (108 ± 68 days). A high-resolution 3D T1 weighted MRI sequence was acquired in a 3 T Philips Ingenia CX scanner (TE/TR = 4.6/9.9 ms, flip angle = 8°; voxel size = 0.75 × 0.75 × 0.75 mm^3^). [^18^F]FDG PET scans were acquired 45 min after the administration of 185 MBq (range 181.3–222 MBq; mean ± SD, 200.83 ± 12.83 MBq) of [^18^F]FDG in a Siemens Biograph mCT scanner, and images were reconstructed using an OSEM3D algorithm (8 iterations, 21 subsets) with PSF + TOF corrections. Centiloid (CL) values [48] were obtained from [^18^F]flutemetamol PET scans to characterize global cerebral Aβ deposition in the participants, as described in [49].

[^18^F]FDG PET scans were first co-registered to the corresponding T1-weighted MRI scans at the MRI subject space. Then, [^18^F]FDG PET scans were normalized to the standard MNI with SPM12 (https://www.fil.ion.ucl.ac.uk/spm/software/spm12/) [50] following a two-step process (Supplementary material). The cerebellar vermis was used as a reference region to calculate standardized uptake value ratio (SUVR) [51]. For the regional analyses, we used the Landau’s meta-ROI, as these areas have been related to early changes in [^18^F]FDG uptake in AD [26]. Unsmoothed images were used to calculate Landau’s meta-ROI SUVR, whereas parametric SUVR images were calculated with the smoothed images for the voxel-wise analyses.

### Statistical analyses

We first compared demographic data by AT stages using an ANOVA for continuous measures and a χ^2^ for categorical data.

#### Comparison between plasma GFAP and CSF GFAP

To investigate the relationship between plasma GFAP and CSF GFAP measures, we performed several analyses. First, we looked at the linear association between both measures adjusting by age, sex, and *APOE-ε4* status (model 1). We repeated this analysis including an interaction term with AT stages to investigate whether this correlation was modified by the stage of the disease (model 2). Three progressive AT stages were considered: A-T-, A + T-, and A + T + .

$$\mathrm{Model}\;1:\mathrm{ Plasma\; GFAP }\sim \mathrm{ age}+\mathrm{ sex}+\mathrm{APOE}-\upvarepsilon 4+\mathrm{ CSF\; GFAP}$$

$$\mathrm{Model}\;2:\mathrm{ Plasma\; GFAP}\hspace{0.17em}\sim \hspace{0.17em}\mathrm{age}\hspace{0.17em}+\hspace{0.17em}\mathrm{sex}\hspace{0.17em}+\mathrm{APOE}-\upvarepsilon 4\hspace{0.17em}+\mathrm{AT}\hspace{0.17em}+\mathrm{CSF\; GFAP}*\mathrm{AT}$$

As additional analyses, we looked for associations between both GFAP biomarkers *(i.e.,* plasma and CSF) and basic characteristics (i.e., age, sex, and *APOE-ε4* status) adjusting by the other relevant covariates [52].For these analyses, we used R (v1.2.5033), and we set the statistical threshold at *p* < 0.05 uncorrected for multiple comparisons.

#### ***Associations with [***^***18***^***F]FDG uptake***

The main objective of our study was to investigate the association between fluid biomarkers of astroglial activation and glucose consumption in the brain. To this aim, we performed two main analyses both at the regional (Landau’s meta-ROI) and at the voxel level. First, we looked at the direct association between each GFAP biomarker and [^18^F]FDG uptake in two independent models (i.e., one for plasma and one for CSF). [^18^F]FDG uptake was set as the dependent variable and GFAP levels as an independent variable, adjusting by age, sex and *APOE-ε4* status (model 3). Second, we repeated these analyses including also an interaction term with AT stages to investigate whether these associations were modified by the AT stage in the *continuum* (model 4).

$$\mathrm{Model}\; 3: {[}^{18}\mathrm{F}]\mathrm{FDG }\sim \mathrm{ age }+\mathrm{ sex }+\mathrm{APOE}-\upvarepsilon 4 +\mathrm{ GFAP\; biomarker}$$

$$\mathrm{Model}\; 4:{[}^{18}\mathrm{F}]\mathrm{FDG} \sim \mathrm{age} + \mathrm{sex} +\mathrm{APOE}-\varepsilon 4 + \mathrm{AT} + \mathrm{GFAP}\; \mathrm{biomarker}*\mathrm{AT}$$

Contrasts were performed on both directions for GFAP biomarker on model 3 and on each of the GFAP biomarker*AT groups comparisons on model 4.

As sensitivity analyses, we repeated model 3 including CSF Aβ42/40 and CSF Aβ42/40 and p-tau as covariates to elucidate whether the correlations observed could be driven by correlations between GFAP levels and Aβ and/or tau. Further, we also repeated models 3 and 4 including only subjects with 6 months or less time difference between GFAP biomarkers obtention and acquisition of the [^18^F]FDG PET image.

SPM12 was used for the voxel-wise analyses, and the statistical significance was set as *p* < 0.005 uncorrected for multiple comparisons with a cluster size of *k* > 100**.**

## Results

A total of 314 participants were included in this study. Their basic demographic characteristics are included in Table 1. In brief, the mean age was 61.1 years old, 62.4% were women, and 54.1% were *APOE-ε4* carriers. Regarding the biomarker-based AT classification, 202 participants were A-T-, 88 were A + T-, and 24 were A + T + . These AT groups were significantly differed in age, percentage of *APOE-ε4* carriers and CL values, showing higher levels in more advanced AT stages (A + T +  > A + T- > A-T-).

**Table 1:** Demographic characteristics by AT stages

	All (*n* = 314)	A-T- (*n* = 202)	A + T- (*n* = 88)	A + T + (*n* = 24)	*p*
Age, years	61.1 (4.7)	60.5 (4.3)	61.7 (5.1)	64.3 (4.7)	< 0.001
Women, *n* (%)	196 (62.4)	130 (64.4)	49 (55.7)	17 (70.8)	0.253
*APOE-ε4* carriers, *n* (%)	170 (54.1)	84 (41.6)	72 (81.8)	14 (58.3)	< 0.001
Plasma GFAP*, pg/ml	136 (55)	121 (42)	153 (66)	201 (53)	< 0.001
CSF GFAP^§^, pg/ml	4303 (2197)	3985 (2045)	4178 (1740)	7415 (2573)	< 0.001
CL	3.0 (17.0)	-4.6 (6.5)	12.5 (17.3)	31.9 (27.1)	< 0.001
Time PET-LP, days	107 (66)	109 (63)	101 (67)	111 (88)	0.571
Time PET-MR, days	145 (71)	146 (66)	140 (71)	150 (98)	0.767

Mean and SD are shown unless otherwise stated. AT groups were derived from previously published thresholds on CSF Aβ42/40 and p-tau [1].*Thirteen values missing. ^§^ Two values missing. Abbreviations: A-T-, Aβ-negative tau-negative; A + T-, Aβ-positive tau-negative; A + T + , Aβ-positive tau-positive; *APOE*, apolipoprotein-E; CL, Centiloids; CSF, cerebrospinal fluid; GFAP, glial fibrillary acidic protein; LP, lumbar puncture; SD, standard deviation

The associations between both plasma and CSF GFAP and demographic characteristics can be found in Fig. S1 and Table S1. In brief, both biomarkers showed a positive correlation with age. Both biomarkers presented significant differences by sex, although in opposite directions, with women having higher plasma GFAP levels, whereas men had higher CSF GFAP levels. Only plasma GFAP levels were associated with *APOE-ε4* status, with carriers presenting higher values.

## Comparison between plasma GFAP and CSF GFAP

**Fig. 1:**
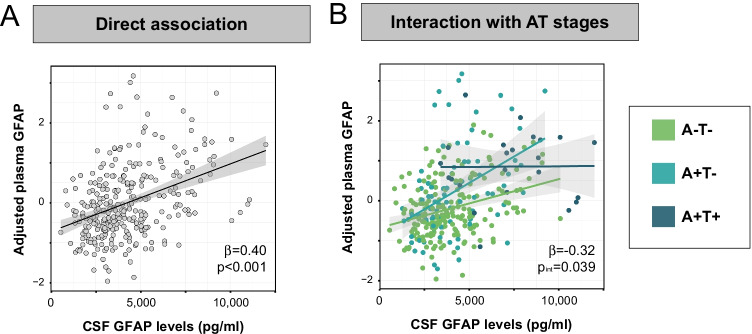
Associations between GFAP biomarkers and CSF Aβ. This figure shows the associations between plasma GFAP and CSF GFAP for all subjects (**A**) and by AT stages (**B**). β and *p* values shown in (**B**) correspond to the interaction effect between tau-negative (A-T- and A + T-) and tau-positive (A + T +) groups. AT groups were derived from previously published thresholds on CSF Aβ42/40 and CSF p-tau [1]. Abbreviations: Aβ, amyloid-β; A-T-, Aβ-negative tau-negative; A + T-, Aβ-positive tau-negative; A + T + , Aβ-positive tau-positive; CSF, cerebrospinal fluid; GFAP, glial fibrillary acidic protein; p-tau, phosphorylated tau

We first compared plasma and CSF levels of GFAP. Plasma GFAP and CSF GFAP were positively correlated (β [95% confidence interval (95%CI)]: 0.40 [0.32,0.49], *p* < 0.001; Fig. 1A). Afterwards, we compared these two biomarkers taking into account the AT stage. We observed that whereas A-T- and A + T- participants showed a positive correlation between plasma and CSF GFAP levels, A + T + participants did not (Fig. 1B). This resulted in a significant difference in the slope between T- (i.e., A-T- and A + T-) and T + (i.e., A + T +) participants (β [95%CI]: − 0.32 [− 0.57, − 0.06], *p* = 0.039).

## ***Correlations with cerebral [***^***18***^***F]FDG uptake***

**Fig. 2:**
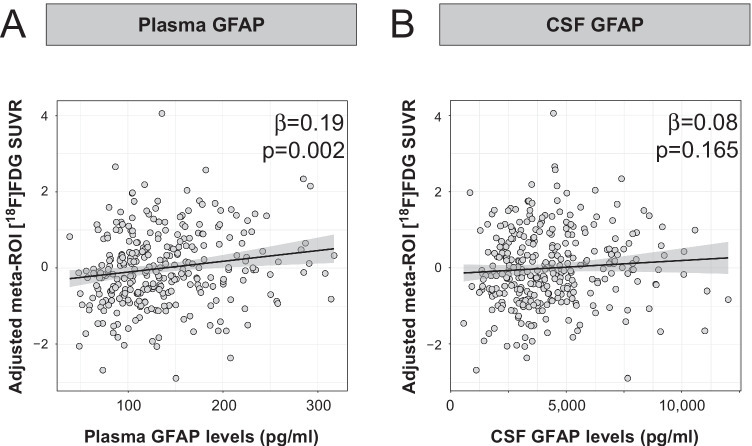
Associations between GFAP biomarkers and [^18^F]FDG uptake in Landau’s meta-ROI. Associations between plasma GFAP (**A**) and CSF GFAP (**B**) and [^18^F]FDG uptake in Landau’s meta-ROI. β and *p* values of the associations with the [^18^F]FDG uptake in the meta-ROI are shown in the plot. Statistical threshold for the meta-ROI analysis was set at *p* < 0.05 uncorrected for multiple comparisons. Abbreviations: CSF, cerebrospinal fluid; [^18^F]FDG, [^18^F]fluorodeoxyglucose; GFAP, glial fibrillary acidic protein

Plasma GFAP (β_plasma_ [95%CI]: 0.19 [0.09, 0.29], *p* = 0.002), but not CSF GFAP (β_CSF_ [95%CI]: 0.08 [− 0.02, 0.18], *p* = 0.165), correlated positively with [^18^F]FDG uptake in the meta-ROI (Fig. 2, Table 2). Sensitivity analyses showed that these associations were not significantly changed when we include CSF Aβ42/40 or CSF Aβ42/40 and CSF p-tau in the models as covariates (Supplementary Table S2 and Figure S2). We also observe that results did not significantly change when we only included subjects with a time difference between [^18^F]FDG PET and GFAP biomarkers drawing equal or below six months (Supplementary Table S3).

**Table 2:** Statistics of the associations between GFAP biomarkers and [^18^F]FDG uptake in Landau’s meta-ROI

	Direct: all subjects		By AT stages						AT interaction	
			A-T-		A + T-		A + T +		A*T- vs A + T +	
	β [95%CI]	p	β [95%CI]	p	β [95%CI]	p	β [95%CI]	p	β [95%CI]	p
Plasma GFAP	**0.19** **[0.09, 0.29]**	**0.002**	**0.20** **[0.08, 0.32]**	**0.008**	**0.28** **[0.10, 0.46]**	**0.013**	− 0.32 [− 0.68, 0.05]	0.154	− 0.33 [− 0.70, 0.04]	0.142
CSF GFAP	0.08 [− 0.02, 0.18]	0.165	0.02 [− 0.10, 0.15]	0.751	**0.28** **[0.10, 0.47]**	**0.013**	− 0.43 [− 0.87, 0.01]	0.106	− **0.45** **[**− **0.74,** − **0.15]**	**0.012**

Linear regression models were used to perform these analyses adjusting by age, sex, and *APOE-ε4* status. A*T- participants (A-T- and A + T-) were selected as the reference group in the interaction with AT analyses. AT groups were derived from previously published thresholds on CSF Aβ42/40 and CSF p-tau [1]. Significant associations (*p* < 0.05) are shown in bold. Abbreviations: Aβ, amyloid-β; A-T-, Aβ-negative tau-negative; A + T-, Aβ-positive tau-negative; A + T + , Aβ-positive tau-positive; *APOE*, apolipoprotein-E; CI, confidence interval; CSF, cerebrospinal fluid; GFAP, glial fibrillary acidic protein; p-tau, phosphorylated tau

**Fig. 3:**
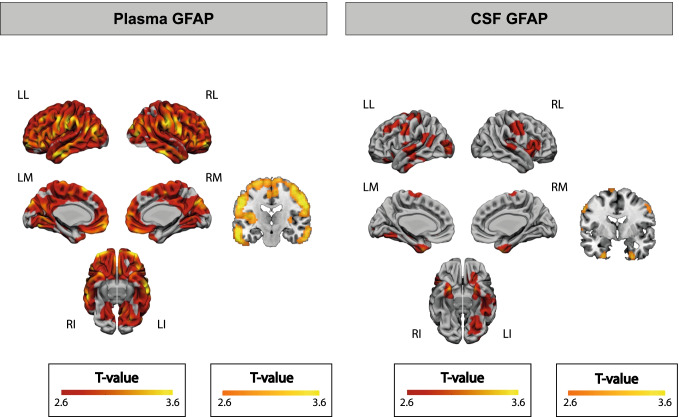
Associations between GFAP biomarkers and [^18^F]FDG uptake at the voxel level. Associations between plasma GFAP (**A**) and CSF GFAP (**B**) and [^18^F]FDG uptake in the whole brain. No negative associations were observed in any area of the brain. Statistical threshold for the voxel-wise analysis was set at *p* < 0.005 uncorrected for multiple comparisons with a cluster size of *k* > 100. Abbreviations: CSF, cerebrospinal fluid; [^18^F]FDG, [^18^F]fluorodeoxyglucose; GFAP, glial fibrillary acidic protein; LI, left inferior; LL, left lateral; LM, left medial; RI, right inferior; RL, right lateral; RM, right medial

At the voxel level, the correlation between plasma GFAP and [^18^F]FDG uptake was observed in almost all cortical brain regions and was absent only in medial temporal and some occipital regions (Fig. 3A). CSF GFAP also correlated positively with [^18^F]FDG uptake but in less spread areas, like temporoparietal areas, including the bilateral temporal poles (Fig. 3B). No negative associations were observed for any of the GFAP biomarkers.

**Fig. 4:**
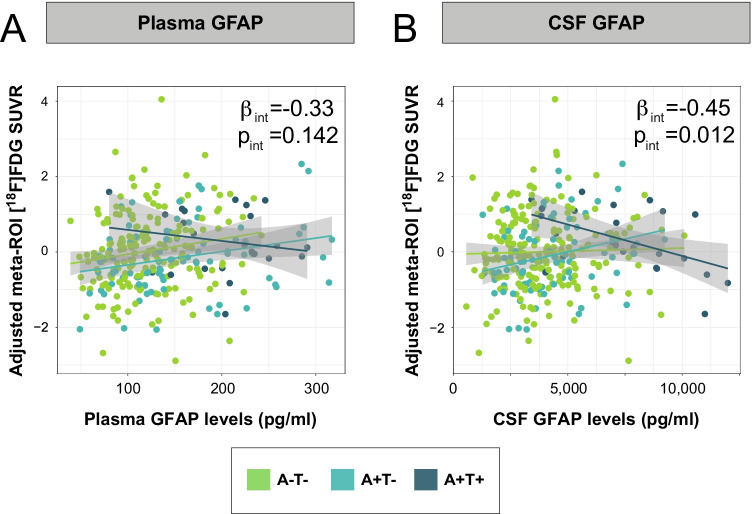
Interaction effect of GFAP biomarkers and AT stages on [^18^F]FDG uptake in Landau’s meta-ROI. Interaction effect of plasma GFAP (**A**) or CSF GFAP (**B**) and AT stages on [^18^F]FDG uptake in Landau’s meta-ROI. β and *p* Values shown in the plot correspond to the interaction effect between tau-negative (A-T- and A + T-) and tau-positive (A + T +) groups. AT groups were derived from previously published thresholds on CSF Aβ42/40 and p-tau [1]. Statistical threshold for the meta-ROI analysis was set *p* < 0.05 uncorrected for multiple comparisons. Abbreviations: Aβ, amyloid-β; A-T-, Aβ-negative tau-negative; A + T-, Aβ-positive tau-negative; A + T + , Aβ-positive tau-positive; CSF, cerebrospinal fluid; [^18^F]FDG, [^18^F]fluorodeoxyglucose; GFAP, glial fibrillary acidic protein; p-tau, phosphorylated tau

**Fig. 5:**
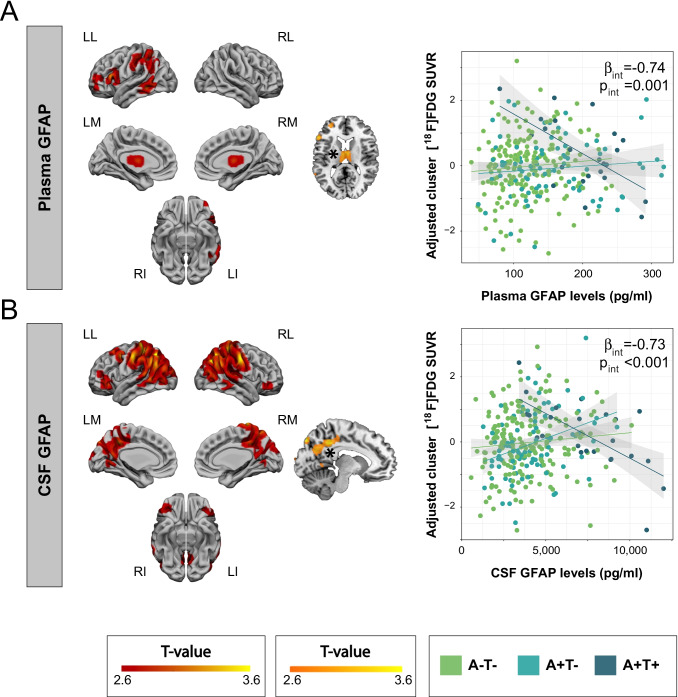
Interaction effect of GFAP biomarkers and AT stages on [^18^F]FDG uptake at the voxel level. Interaction effect of plasma GFAP (**A**) or CSF GFAP (**B**) and AT stages on [^18^F]FDG uptake in the whole brain. The first (projection) and second (slice) columns show the areas where A-T- and A + T- participants have a significantly different association than A + T + participants between each GFAP biomarker and [^18^F]FDG uptake. The third column depicts the specific association between adjusted [^18^F]FDG uptake in the cluster marked with an asterisk (*) and each of the GFAP biomarkers by AT stages. Scatter plots of the other clusters can be found in the Supplementary Material. β and *p* values shown in the plot correspond to the interaction effect between tau-negative (A-T- and A + T-) and tau-positive (A + T +) groups. AT groups were derived from previously published thresholds on CSF Aβ42/40 and p-tau [1]. Statistical threshold for the voxel-wise analysis was set at *p* < 0.005 uncorrected for multiple comparisons with a cluster size of *k* > 100 and *p* < 0.05 uncorrected for multiple comparisons for the cluster analysis. Abbreviations: Aβ, amyloid-β; A-T-, Aβ-negative tau-negative; A + T-, Aβ-positive tau-negative; A + T + , Aβ-positive tau-positive; CSF, cerebrospinal fluid; [^18^F]FDG, [^18^F]fluorodeoxyglucose; GFAP, glial fibrillary acidic protein; LI, left inferior; LL, left lateral; LM, left medial; p-tau, phosphorylated tau; RI, right inferior; RL, right lateral; RM, right medial

We observed that the interaction between T- and T + participants was significant in the meta-ROI with CSF GFAP but not with plasma GFAP (Fig. 4 and Table 2) so that in T + participants, the association between GFAP biomarkers and [^18^F]FDG uptake became negative in these areas. In the voxel-wise analysis, this interaction was significant in left temporo-parietal areas and bilateral thalamus with plasma GFAP with a similar behavior (Fig. 5A). This significant interaction was also observed with CSF GFAP in temporo-parietal areas, although more widespread, including medial regions such as precuneus and posterior cingulate (Fig. 5B). Only one representative cluster scatter plot is shown in Fig. 5 per biomarker, but we present all the others in the Supplementary Material (Fig. S3). The inverse contrast did not present any significant result with any of the GFAP markers. Additionally, we present mean [^18^F]FDG uptake maps for each of the AT groups in the Supplementary Material (Fig. S4).

## Discussion

The main finding of this study is that higher plasma GFAP is strongly associated with higher global cerebral glucose consumption early in the Alzheimer’s *continuum*. Interestingly, this association was more widespread than the one found for its CSF counterpart. The regional cerebral pattern of the association with plasma GFAP included, but was not restricted to, areas of early amyloid deposition, such as the posterior and anterior cingulate cortices. Importantly, these associations were independent of Aβ pathology, as measured by CSF Aβ42/40, and tau pathology, as measured by CSF p-tau. Nonetheless, in specific areas showing hypometabolism in AD [26], such as lateral parietal and middle frontal lobe, this positive association between plasma GFAP and [^18^F]FDG uptake occurred in the group of individuals who were still tau-negative (T-) but the correlation turned negative in the group of tau-positive (T +) individuals. To our knowledge, this is the first report of associations between plasma GFAP and [^18^F]FDG PET in the Alzheimer’s *continuum*. Our findings suggest that higher astrocytic reactivity, probably in response to early AD pathological changes, is related to significantly higher glucose consumption. However, the association between astrocytic reactivity and glucose consumption seems to uncouple with the onset of tau pathology, maybe due to a failure in sustaining such elevated energetic demands.

Studying the relationship between metabolic demand and astrocytic reactivity in early preclinical AD stages is very relevant because, under physiological conditions, the brain accounts for 20–25% of overall glucose-derived energy [33, 53], half of which is presumably consumed by astrocytes [35–37]. Such energetic consumption is continuous, and its deprivation, even for short durations, can result in neuronal damage and death [54]. Therefore, failure to sustain this metabolic demand already in preclinical AD stages may have important deleterious consequences later in the course of the disease.

Previous literature examined astrocytic reactivity and glucose consumption in sporadic and autosomal dominant AD patients with [^11^C]deuterium-L-deprenyl ([^11^C]DED) PET. [^11^C]DED is a PET radiotracer that has been used to visualize reactive astrocytes and that colocalizes with GFAP immunohistochemistry in autoradiographic post mortem studies [55]. In these reports, higher [^11^C]DED binding was found in presymptomatic mutation carriers and sporadic Aβ + MCI, but not in sporadic AD dementia patients [56, 57]. This result was suggestive of an early increase of astrocytosis in presymptomatic mutation carriers that decreased in more advanced stages. While no associations were found between [^11^C]DED and [^18^F]FDG uptake at baseline, longitudinal follow-up revealed that uptake of both tracers declined as the mutation carriers converted from presymptomatic to prodromal AD, especially in the precuneus as well as in frontal and cingulate cortices [58]. This suggests that the non-monotonic astrocytic response to early AD pathological changes had a metabolic correlation [59]. Our findings are in close agreement with these previous studies with [^11^C]DED and [^18^F]FDG PET, showing also a direct link between astrocytic response and metabolism. However, the absence of Aβ + cognitively unimpaired participants in these previous studies prevented the extrapolation of their findings to the sporadic preclinical Alzheimer’s *continuum*.

In this regard, the main contribution of our study to the existing literature is the confirmation of an association between reactive astrogliosis and cerebral glucose consumption early in the Alzheimer’s *continuum* that, as shown here, is inverted in participants with tau pathology in preclinical stages in specific regions like parietal and middle frontal lobe. This change of behavior may be related to a phenotypic change in astrocytes under pathological conditions. In a physiological situation, astrocytes significantly contribute to [^18^F]FDG uptake by the activation of astroglial glutamate transport [39]. However, in the presence of tau pathology, reactive astrocytes expressing higher levels of GFAP consistently express lower levels of genes associated with glutamate/GABA homeostasis [60]. In turn, a breakdown of normal astrocyte function leads to impaired clearance of amyloid and proinflammatory cytokine release, which eventually might lead to neurodegeneration [6]. In this regard, it is worth noting that the negative association between [^18^F]FDG and CSF GFAP, and in a lesser extent plasma GFAP, in the A + T + group was found in typical areas of early AD-related hypometabolism, such as the precuneus and parieto-temporal areas [26]. Therefore, it could be thought that such stage-dependent glucose metabolic changes associated with reactive astrocytes might contribute to the hypometabolism observed in clinical AD stages, which is thought to reflect synapse loss and neurodegeneration.

Interestingly, we also found this change of behavior in the thalami when looking at the association between plasma GFAP and [^18^F]FDG. Although this is not a typical AD region, we and others have previously found some metabolic alterations in the thalami in the early stages of the Alzheimer’s *continuum*. For instance, Johnson and colleagues reported increased [^18^F]FDG uptake in the thalami in cognitively unimpaired Aβ + subjects and participants with intermediate levels of amyloid-β compared to Aβ- participants [61]. In a more recent study, we also found increased glucose consumption in cognitively unimpaired participants, which we were able to relate to increased CSF core AD biomarkers as well as increased CSF markers of neuroinflammation, including GFAP and YKL-40 [62]. Thus, it seems that glucose consumption in the thalami may be especially altered in these early stages of AD, presumably due to an increase of astrocytic reactivity in relation to early amyloid-β and tau pathologies.

A surprising finding was that the association with [^18^F]FDG was more widespread with plasma than with CSF GFAP and only significant in AD-related regions in the case of plasma GFAP. In addition, we and others have shown that plasma GFAP changes with Aβ pathology were stronger with plasma than CSF GFAP [21, 22]. Therefore, our findings also support the idea that plasma GFAP may be an earlier marker than its CSF counterpart. The question that then arises is whether plasma and CSF GFAP come, at least partially, from different sources. The fact that plasma and CSF GFAP have a significant but mild correlation also favors this idea. A preferential release of GFAP to blood flow in response to Aβ pathology might be explained since astrocytes, as part of the blood brain barrier (BBB), have end-feet positioned at the intraparenchymal capillaries that allow them to regulate the delivery of oxygen and glucose to neurons [33, 54]. It could be argued that GFAP found in blood better reflects early BBB dysfunction in response to Aβ pathology than CSF GFAP through direct release of the protein from these end-feet into the bloodstream. This is also supported by the rapid release of GFAP in blood after other types of BBB dysfunction such as traumatic brain injury [63].

It should also be noted that in another recent study with the same participants, we also investigated the association between [^18^F]FDG uptake and CSF YKL-40, another marker related to astrogliosis [62]. As previously stated, in that study, we found that a combination of increased core and neuroinflammation CSF markers were related to increased glucose metabolism in thalami but also in the striatum, and the inferior and transverse temporal gyri. Interestingly, this combination of increased CSF markers was also related to larger grey matter volumes in bilateral insula, bilateral inferior temporal, bilateral supramarginal, and inferior occipital cortices, which suggests that inflammation processes related to early amyloid and tau alterations have an effect not only in brain metabolism but also in its structure. Altogether, it warrants for further investigation in other cohorts to try to decipher the specific relationships between astrocytic reactivity and metabolic and structural brain alterations in early stages of the Alzheimer’s *continuum*.

Our study is observational and cross-sectional. Therefore, we cannot disentangle any causal relationships. It could be thought that astrocytes react to early Aβ dysmetabolism, but others have postulated that astrocytic metabolic dysfunction might trigger the amyloidogenic processing of the amyloid precursor protein [64]. Still, our findings support conducting longitudinal and interventional studies to better understand the causal inter-relationships among these alterations early in the Alzheimer’s *continuum*. A better understanding of the pathophysiological pathways affected in preclinical stages is fundamental for the rational design of preventive interventions. Another limitation in our study is that we assume GFAP to be a marker of reactive astrocytes. In this regard, it must be noted that GFAP is not exclusive to astrocytes in the central nervous system and that not all astrocytes produce GFAP [65]. Still, GFAP is the best well-established and commonly used astrocytic marker in the literature [11]. Finally, since astrocytes are not the only metabolically active non-neuronal cells in the brain, it could be thought that the observed increase in glucose consumption is driven by other processes such as microglial activation. In this regard, a recent study shown that microglial activation can also be a significant contributor to the [^18^F]FDG PET signal [66]. Given that astrocytes can directly trigger microglial activation [67], it could be possible that the increase of glucose consumption is indirectly mediated or significantly contributed by microglial activation. Nonetheless, in a recent study of our group with the same participants of this study, we did not find the same kind of relation with sTREM2 [62], a marker of microglial activation, and [^18^F]FDG uptake as the one found here with plasma or CSF GFAP. Thus, this result does not support to the microglial involvement in the association here presented.

Our study has several strengths. The presence of biomarkers of Aβ and tau pathology has enabled us to stage a large cohort of cognitively unimpaired individuals within the AT framework. In addition, we used the same precise and validated assay to measure GFAP both in plasma and CSF, thus avoiding potential methodological confounders associated with using different analytical platforms in the two biofluids. This assay is commercially available, and, therefore, other research groups can replicate our results in their cohorts. Finally, we could compare head-to-head the associations between [^18^F]FDG uptake and CSF and plasma GFAP given that we measured both fluid biomarkers moment in the same time.

In summary, we found that higher plasma GFAP, a marker of reactive astrogliosis, is associated with widespread higher cerebral glucose metabolism as measured with [^18^F]FDG in the preclinical Alzheimer’s *continuum*. Of note, the correlation with glucose metabolism was also observed with GFAP levels measured in the CSF, although less widespread and in different regions. This finding suggests that early Alzheimer’s pathologic changes trigger an astrocytic reaction that is linked to greater energetic demand. With the onset of tau pathology, this coupling between astrogliosis and cerebral glucose consumption is reversed in regions of early AD-related hypometabolism. This suggests a failure to sustain such an elevated energetic consumption due to a disruption of glucose metabolism in astrocytes, already in preclinical AD stages. Our findings contribute to a better understanding of the metabolic requirements of the neuroinflammatory response to early AD pathology, which will be useful for the rational design of preventive interventions targeting inflammatory mechanisms and/or energetic dysfunction in preclinical stages of AD.

## Supplementary Information

Below is the link to the electronic supplementary material.

Supplementary file1 (DOCX 54.4 MB)

## Data Availability

The datasets generated during the current study are available from the corresponding author on reasonable request.
