# Supplementary material for: Reactive astrogliosis is associated with higher cerebral glucose consumption in the early Alzheimer’s continuum
Source: Eur J Nucl Med Mol Imaging. 2022 Jul 18;49(13):4567–79. doi: 10.1007/s00259-022-05897-4 (PMC9606048; doi:10.1007/s00259-022-05897-4)
Supplement: Supplementary file 1 — Supplementary file1 (DOCX 54.4 MB) [file 259_2022_5897_MOESM1_ESM.docx]

# INCREASED PLASMA GFAP IS ASSOCIATED WITH HIGHER CEREBRAL GLUCOSE CONSUMPTION IN THE EARLY ALZHEIMER’S *CONTINUUM*

Gemma Salvadó^*^, Marta Milà-Alomà^*^, Mahnaz Shekari, Nicholas J. Ashton, Grégory Operto, Carles Falcon, Raffaele Cacciaglia, Carolina Minguillon, Karine Fauria, Aida Niñerola-Baizán, Andrés Perissinotti, Andréa L. Benedet, Gwendlyn Kollmorgen, Ivonne Suridjan, Norbert Wild, José Luis Molinuevo, Henrik Zetterberg, Kaj Blennow, Marc Suárez-Calvet, Juan Domingo Gispert for the ALFA Study

^*^ These authors have equally contributed to this work

# **Methods**

## Image acquisition and processing

First, MRI images were normalized to the MNI space using the *segment* function. This transformation was used to move the [^18^F]FDG PET scans to the MNI. In this space, we created an [^18^F]FDG PET template with all the images available. Finally, we moved the original [^18^F]FDG PET scans to this template in the MNI space with the *old normalize* function. [^18^F]FDG uptake in the brain was normalized to the cerebellar vermis [1]. Spatially normalized [^18^F]FDG scans were also smoothed by a three-dimensional Gaussian kernel of 12 mm full width at half maximum (FWHM) for the voxel-wise analysis.

# **Results**


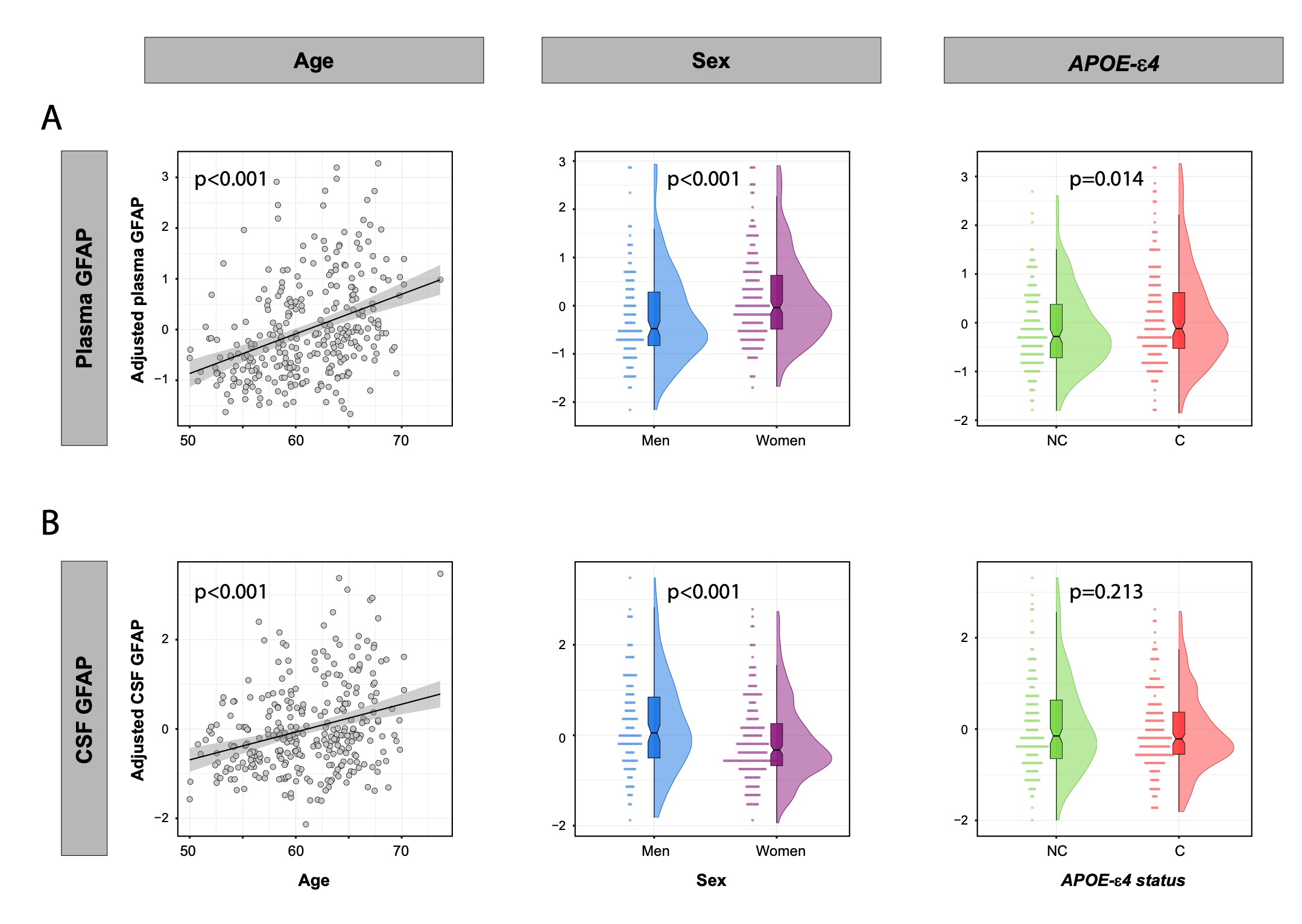


**Figure s1 Associations between GFAP biomarkers and demographics**

Associations between plasma GFAP (A) or CSF GFAP with age (first column), sex (second column) and *APOE-ε4* status (third column). P-values of the associations are shown in the plots.

Abbreviations: *APOE*, apolipoprotein-E; C, carrier; CI, confidence interval; CSF, cerebrospinal fluid; GFAP, glial fibrillary acidic protein; NC, non-carrier.

|  | Age | | Sex | | *APOE-ε4* | |
| --- | --- | --- | --- | --- | --- | --- |
|  | β [95%CI] | p | β [95%CI] | p | β [95%CI] | p |
| Plasma GFAP | 0.37  [0.28,0.45] | <0.001 | 0.39  [0.21,0.57] | <0.001 | 0.26  [0.09,0.44] | 0.014 |
| CSF  GFAP | 0.29  [0.21,0.38] | <0.001 | -0.38  [-0.56,-0.19] | <0.001 | -0.13  [-0.31,0.04] | 0.213 |

**Table s1 Statistics of the associations between GFAP biomarkers and demographics**

Men and *APOE-ε4* non-carriers were selected as reference groups.

Abbreviations: *APOE*, apolipoprotein-E CI, confidence interval; CSF, cerebrospinal fluid; GFAP, glial fibrillary acidic protein.

|  | Adjusted for Aβ | | Adjusted for Aβ and tau | |
| --- | --- | --- | --- | --- |
|  | β [95%CI] | p | β [95%CI] | p |
| Plasma GFAP | 0.23 [0.13, 0.34] | <0.001 | 0.23 [0.12, 0.34] | <0.001 |
| CSF GFAP | 0.09 [-0.01, 0.19] | 0.156 | 0.08 [-0.04,0.20] | 0.275 |

**Table s2 Associations between GFAP biomarkers and [^18^F]FDG uptake in the meta-ROI after adjusting for Aβ and tau**

Statistical threshold was set p<0.05 uncorrected for multiple comparisons.

Abbreviations: Aβ, amyloid-β; CSF, cerebrospinal fluid; GFAP, glial fibrillary acidic protein; ROI, region of interest.

**Figure s2 Associations between GFAP biomarkers and [^18^F]FDG uptake after adjusting for Aβ and tau at the voxel level**

Associations between plasma GFAP (A, C) or CSF GFAP (B, D) and [^18^F]FDG uptake at the voxel level after adjusting for CSF Aβ42/40 (first column) or adjusting for CSF Aβ42/40 and CSF p-tau (second column). Statistical threshold was set p<0.005 uncorrected for multiple comparisons with a cluster size of k>100.

Abbreviations: Aβ, amyloid-β; CSF, cerebrospinal fluid; GFAP, glial fibrillary acidic protein; p-tau, phosphorylated tau.

|  | Direct association | | Interaction with AT stages | |
| --- | --- | --- | --- | --- |
|  | β [95%CI] | p | β [95%CI] | p |
| Plasma GFAP | 0.19  [0.08, 0.30] | 0.005 | -0.34  [-0.73, 0.05] | 0.150 |
| CSF GFAP | 0.09  [-0.01, 0.20] | 0.156 | -0.33  [-0.65, -0.02] | 0.079 |

**Table s3 Associations between GFAP biomarkers and [^18^F]FDG uptake in the meta-ROI with data acquired in less than 6 months difference**

Only participants that had a time difference below a time difference between [^18^F]FDG PET and GFAP biomarkers drawing equal or below six months. In the interaction analysis with AT stages, we compared A*T- (Aβ negative tau negative and Aβ positive tau positive) against A+T+ group.

Abbreviations: Aβ, amyloid-β; CSF, cerebrospinal fluid; GFAP, glial fibrillary acidic protein; ROI, region of interest.

**
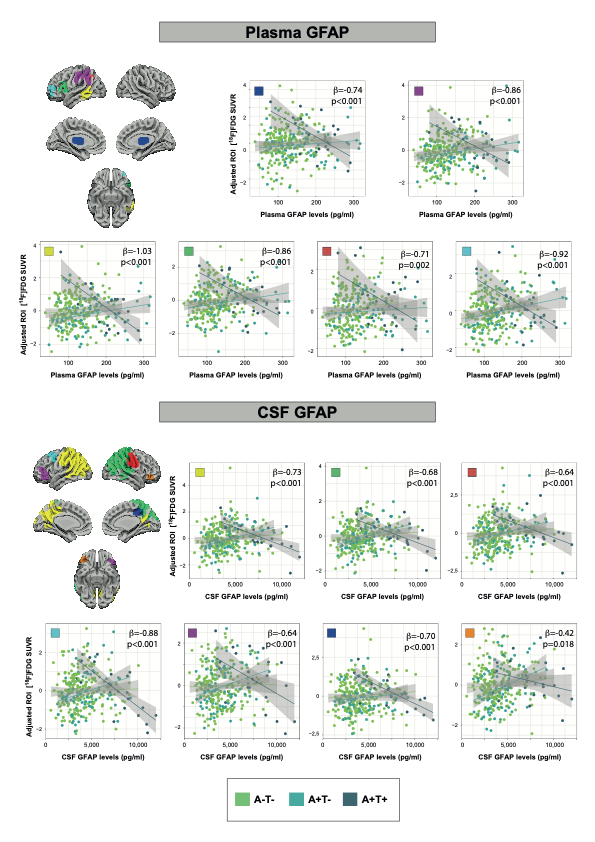
**

**Figure s3 Interaction effect of GFAP biomarkers and AT stages on [^18^F]FDG uptake at the voxel level**

Scatter plots of all significant clusters are shown here. The correspondence between clusters and scatter plots are depicted by colored squares at the upper left corner of each plot. β’s and p-values shown in the plot correspond to the interaction effect between tau-negative (A-T- and A+T-) and tau-positive (A+T+) groups. AT groups were derived from previously published thresholds on CSF Aβ42/40 and p-tau [1]. Statistical threshold for the voxel-wise analysis was set at p<0.005 uncorrected for multiple comparisons with a cluster size of k>100 and p<0.05 uncorrected for multiple comparisons for the cluster analysis.

Abbreviations: Aβ, amyloid-β; A-T-, Aβ-negative tau-negative; A+T-, Aβ-positive tau-negative; A+T+, Aβ-positive tau-positive; CSF, cerebrospinal fluid; [^18^F]FDG, [^18^F]fluorodeoxyglucose; GFAP, glial fibrillary acidic protein; LI, left inferior; LL, left lateral; LM, left medial; p-tau, phosphorylated tau; RI, right inferior; RL, right lateral; RM, right medial.

**Figure s4 Mean [^18^F]FDG uptake per AT group**

Abbreviations: A-T-, Aβ-negative tau-negative; A+T-, Aβ-positive tau-negative; A+T+, Aβ-positive tau-positive;

# **References**

[1] Rasmussen JM, Lakatos A, van Erp TGM, Kruggel F, Keator DB, Fallon JT, et al. Empirical derivation of the reference region for computing diagnostic sensitive  ^18^fluorodeoxyglucose ratios in Alzheimer’s disease based on the ADNI sample. Biochimica et Biophysica Acta 2012;1822:457–66. https://doi.org/10.1016/j.bbadis.2011.09.008.

[2] Milà-Alomà M, Salvadó G, Gispert JD, Vilor-Tejedor N, Grau-Rivera O, Sala-Vila A, et al. Amyloid-β, tau, synaptic, neurodegeneration and glial biomarkers in the preclinical stage of the Alzheimer’s continuum. Alzheimer’s and Dementia 2020:1–14. https://doi.org/10.1002/alz.12131.
